# Effect of Fibers on Durability of Concrete: A Practical Review

**DOI:** 10.3390/ma13204562

**Published:** 2020-10-14

**Authors:** Suvash Chandra Paul, Gideon P.A.G. van Zijl, Branko Šavija

**Affiliations:** 1 Department of Civil Engineering, International University of Business Agriculture and Technology, Dhaka 1230, Bangladesh; suvashpl@iubat.edu; 2 Department of Civil Engineering, Stellenbosch University, Private Bag X1, Matieland 7602, Stellenbosch, South Africa; gvanzijl@sun.ac.za; 3 Microlab, Faculty of Civil Engineering and Geosciences, Delft University of Technology, 2628CN Delft, The Netherlands

**Keywords:** fiber reinforced concrete (FRC), durability, FRC applications, case studies

## Abstract

This article reviews the literature related to the performance of fiber reinforced concrete (FRC) in the context of the durability of concrete infrastructures. The durability of a concrete infrastructure is defined by its ability to sustain reliable levels of serviceability and structural integrity in environmental exposure which may be harsh without any major need for repair intervention throughout the design service life. Conventional concrete has relatively low tensile capacity and ductility, and thus is susceptible to cracking. Cracks are considered to be pathways for gases, liquids, and deleterious solutes entering the concrete, which lead to the early onset of deterioration processes in the concrete or reinforcing steel. Chloride aqueous solution may reach the embedded steel quickly after cracked regions are exposed to de-icing salt or spray in coastal regions, which de-passivates the protective film, whereby corrosion initiation occurs decades earlier than when chlorides would have to gradually ingress uncracked concrete covering the steel in the absence of cracks. Appropriate inclusion of steel or non-metallic fibers has been proven to increase both the tensile capacity and ductility of FRC. Many researchers have investigated durability enhancement by use of FRC. This paper reviews substantial evidence that the improved tensile characteristics of FRC used to construct infrastructure, improve its durability through mainly the fiber bridging and control of cracks. The evidence is based on both reported laboratory investigations under controlled conditions and the monitored performance of actual infrastructure constructed of FRC. The paper aims to help design engineers towards considering the use of FRC in real-life concrete infrastructures appropriately and more confidently.

## 1. Introduction

A concrete structure may be exposed to a variety of environmental conditions throughout its service life. The durability of a concrete structure is therefore defined by its ability to withstand these exposure conditions without major repair or rehabilitation. It has long been believed that concrete is an inherently durable material, which can last many decades or even centuries with no or very little maintenance. However, the relatively low tensile strength and (quasi)-brittle behavior in tension necessitate the use of reinforcement (typically steel rebars or fibers) in most structures. While steel rebars are still predominantly applied, the use of fibers has seen much development in the past decades. Various types of micro and macro fibers have been used as a secondary reinforcement in concrete.

Fiber reinforced concrete (FRC) is a special class of concrete which incorporates fibers in the formulation to enhance its resistance to (tensile) loads. Different classes of FRC with varying advantages have been developed. Fibers with different cross sections (flat, circular, rectangular, etc.) have been used. Traditionally, fibers have been classified in terms of materials: commonly used are metallic, glass, synthetic, and natural fibers. A further distinction can be made based on the fiber size: we differentiate between micro (shorter than 20 mm with an equivalent diameter of 5–200 μm) and macro fibers (typically 20–80 mm long with a length to diameter ratio of 40–120). Physical properties of commonly used fiber types are summarized in Table 1.

**Figure 1:**
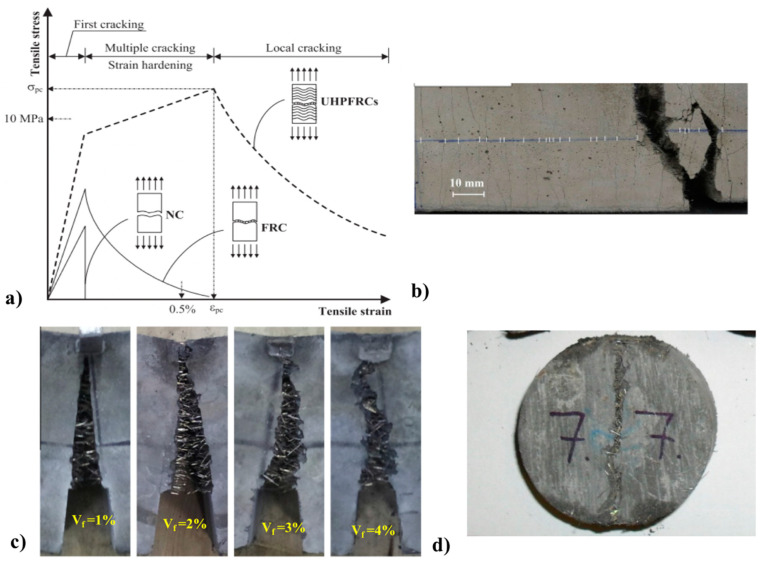
Benefit of using fiber in concrete (**a**) a comparison of different types of concrete in tensile stress and strain, (**b**) cracks in SHCC, (**c**) crack mouth opening in notched beam for different volume of steel fiber fibers and (**d**) FRC damaged in a splitting test [6,7,8,9].

Continued development of fibers has resulted in many classes of advanced FRC’s. Engineered, strain hardening cementitious materials (ECC/SHCC) [1,2,3] are characterized by high tensile ductility and multiple, finely spaced cracks controlled by fiber-bridging. On the other hand, ultra-high tensile and compressive strength are the main characteristics of ultra-high performance fiber-reinforced concrete (UHPFRC) [4,5]. The behavior of FRC depends on many factors, such as fiber aspect ratio (length/diameter), modulus of elasticity, volume percentage included in the composite, fiber orientation, concrete workability through its influence on fiber dispersion and orientation, and size of coarse aggregates. Therefore, for exploiting the maximum benefits of using fibers, all these factors must be considered and incorporated carefully in the concrete material and structural design. Some benefits of using different types of FRC are depicted in Figure 1 [6,7,8,9]. Compared to normal concrete (NC) and FRC, the remarkable material properties of UHPFRC lead to high tensile strain and strength capacities and the development of a pseudo-plastic phase (strain hardening) prior to concrete softening, which is responsible to high energy absorption (toughness) before fracturing [10]. The fracture energy of UHPFRC can also be significantly higher (about five times) than the FRC [10].

The optimum fiber content in FRC depends on the FRC class, and the fiber type and aspect ratio. For micro-fiber such as polyvinyl alcohol (PVA) and polyethylene (PE), the optimum content was found to be in a range of 1–2% in SHCC [11,12]. In the case of steel fibers, this range was found to be between 1.5 and 2.5% in HPFRC [13,14,15]. The variation in the optimum level between studies could be explained by the different aspect ratio of fibers used. Similarly, for macro fibers of PP (length up to 70 mm), this optimum was found to be between 0.5 to 1% for strain-softening FRC [16,17], while up to 0.5% of steel macro-fiber is typical in traditional FRC ground slabs [18].

Due to its numerous advantages—good tensile strength, ductility, fatigue resistance—FRC’s have been used in a wide range of applications, including pavements, industrial floors, tunnel linings, slope stabilization, and impact resistant structures, among others [19]. The appropriate use of FRC may increase the durability and service life of a structural element, thereby also reducing the overall environmental impact of the element over its entire lifecycle. Since FRC has high toughness and resistance to impact, its use may be beneficial in the precast industry due to reduced susceptibility to damage during transport and handling. Furthermore, the use of steel fibers has been shown to result in higher resistance to shear failure of reinforced concrete beams, thereby reducing the need for stirrups [20,21,22]. In compressed elements, conventional reinforcement (i.e., rebars) may be replaced by fiber reinforcement [23]. In beam elements, fiber reinforced concrete has been also used in hybrid concrete structures as a surface layer for crack width control [24,25]. Fiber reinforced concretes of different types have been successfully used as repair materials in several real-life projects including bridge decks, concrete dams, tunnels, coupling beams in high-rise buildings, in the USA, Japan, and Germany [26,27].

FRC is also used for strengthening of structures sensitive to earthquakes [28]. The higher ductility and fracture behavior of FRC can also reduce the risk of damage to the RC structure due to seismic loading. Due to its better mechanical and durability performance, FRC’s have also become popular for underground structures in seismically active areas [29]. Several studies also focused on the sensitivity analysis of the structural response during seismic loading with respect to the variation of mechanical parameters [30,31].

For practical use of fiber reinforced concrete, it is important to understand its long-term performance in different environmental conditions. Compared to plain concrete, the field of fiber reinforced concrete is less mature. Consequently, significant research efforts have been made in recent years to increase our understanding of the durability of FRC. This article aims to review the existing literature related to durability of FRC in terms of different deterioration mechanisms, including chloride and carbonation-induced corrosion, freeze/thaw cycles, and alkali-silica reaction. Some practical applications are also discussed. This review can form a basis for the practical use of FRC in infrastructures, as well as provide researchers with background before embarking on research in the field of FRC durability.

## 2. Deterioration Processes Affecting Fiber Reinforced Concrete

In general, deterioration of concrete may be caused by a variety of physical, chemical, or mechanical processes which commonly act in combination [32]. One of the most common deterioration mechanisms affecting reinforced concrete infrastructures is corrosion of steel reinforcement. Corrosion is an electrochemical process in which the charge (electrons and ions) flow from the anode to the cathode. In general, a passive film forms spontaneously on the steel surface in the alkaline environment of hydrating concrete [33], protecting it from corrosion. However, this passive film can break down due to chloride ingress [34,35] or carbonation [36,37], leading to active corrosion [38,39,40]. Furthermore, in concrete the corrosion process (both the initiation [41] and the propagation period [42]) is strongly influenced by presence of cracks. As the cracking process in FRC’s is fundamentally different than in plain concrete, it is reasonable to expect that the deterioration processes are also different. A brief overview of existing literature related to the influence of fibres on the corrosion of steel reinforcement in cracked concrete is given in Table 2, while details are discussed in the coming sections. 

### 2.1. Fiber Reinforced Concrete Subjected to Chlorides

As described, chloride ions can cause corrosion of reinforcing steel in concrete. While sometimes present in the concrete mix—as part of contaminated aggregate or used as set accelerators in the past—typically chlorides from the environment penetrate the concrete cover and slowly reach the steel. The main consequence of chloride ingress is reinforcement corrosion. Pitting is a typical form of chloride induced corrosion [39]. Since a certain amount of chlorides at the level of the reinforcement is needed to initiate corrosion, the period needed for corrosion initiation depends to a large extent on the integrity of the concrete cover. In addition, chlorides may be present in concrete either as free or bound chloride, and it is only the free chlorides that contribute to reinforcement corrosion. Chloride binding to the cementitious matrix is a complex process and depends on many factors [48]. Presence of chemical compounds of cement such as tri-calcium aluminate (C_3_A) and tetra-calcium alumina ferrite (C_4_AF) together with the bound chloride ions form Friedel’s salt, which has a less porous structure and slows down the transport of chloride ions [49]. Overall, the chloride binding capacity of concrete depends on C_3_A, C_4_AF, tri-calcium silicate (C_3_S), water/cement ratio, of which C_3_A has the most dominant effect [50]. Therefore, chloride binding in FRC will depend mainly on the matrix constituents.

As already mentioned, issues related to chloride ingress may be even more pronounced in the presence of cracks. Therefore, in this section, the focus is mostly on studies related to performance of FRC under coupled effects of cracking and chloride ingress.

Chloride resistance of uncracked steel fiber FRC and RC specimens was investigated by Abbas et al. [51] through rapid chloride migration test (RCPT) as recommended by ASTM C1202-10. The chloride diffusion coefficient was also calculated using Fick’s second law. It was found that the chloride diffusion coefficient and the charge passed through FRC samples were lower than that of RC. This was attributed to the ability of FRC to arrest micro-cracks by the fibers (mixture of steel and PP) during curing and handling [52]. In general, the onset of cracking caused by reinforcement corrosion is delayed by the fiber reinforcement [53,54,55]. In this process, the fiber-matrix interface may play an important role in uncracked FRC. Generally, better fiber matrix interface reduces the expansion of corrosion products in the steel bars. It is considered that the fiber-matrix interface is denser and more uniform due to rich calcium hydroxide than the interface between conventional steel rebars and the matrix, preventing the deleterious solutes entering the FRC [56,57]. The high content of calcium hydroxide present in the fiber–matrix interface may increase chloride binding [58]. Nevertheless, the excessive damage in the fiber–matrix interface would result in a progressive and localized reduction of the fiber cross-section due to corrosion.

**Figure 2:**
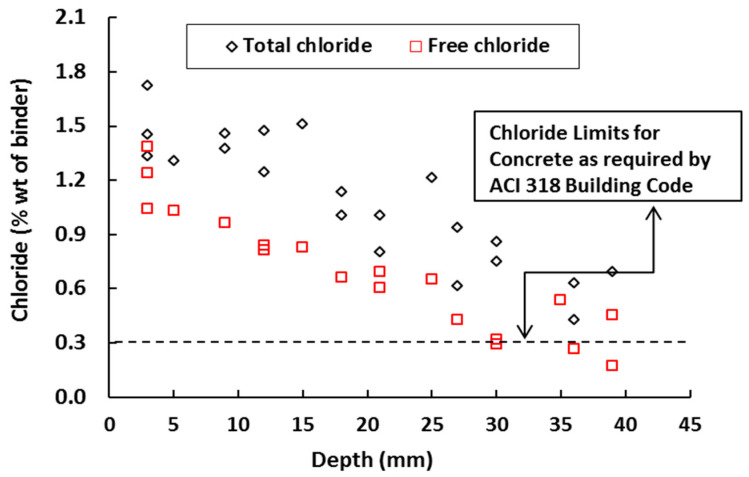
Chloride penetration at different depths in multiple cracked SHCC specimens (average crack width below 50 µm) with 2% PVA fibers. Black diamonds and red squares refer to total and free chloride, respectively.

**Figure 3:**
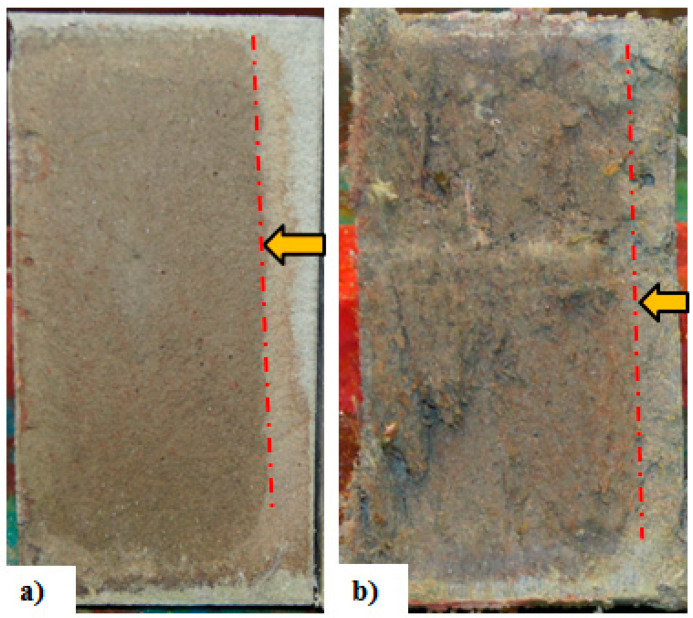
Chloride penetration depth in (**a**) mortar and (**b**) SHCC specimens after a rapid chloride migration test (note: arrows represent the chloride penetration direction).

Excellent crack bridging capacity and formation of multiple fine cracks in SHCC result in good resistance against chloride penetration, which can delay the corrosion initiation process in reinforced concrete [12,44]. Figure 2 shows the penetration of both total and free chloride at multiple cracked SHCC specimens (average crack widths below 50 µm) with 2% PVA fibers under an accelerated chloride induced corrosion test [59,60]. Note that the dashed line in the figure shows the critical chloride content for concrete (minimum compressive strength 17 MPa) without any fibers exposed to moisture but not to external sources of chlorides as recommended by ACI 318 Building Code [61]. In Figure 3, the chloride penetration depth of (uncracked) mortar and SHCC (2% PVA fibers) specimens are shown after the rapid chloride migration test (NT Build 492). In the uncracked state, slightly lower chloride penetration was found in SHCC specimens compared with mortar specimens.

Resistance to steel corrosion in high-performance fiber-reinforced cementitious composite (HPFRCC) made with 1.5% by volume PE fibers with bending cracks was studied by Miyazato & Hiraishi [45]. In their study, steel reinforced specimens made using both HPFRCC and standard mortar were first subjected to bending at constant load of 20 kN to create cracks. Then, the cracked faces of the specimens were exposed to weekly wetting/drying cycles with a 3% NaCl solution for 28 days (2 days wetting, 5 days drying). Linear polarization resistance was used to measure the corrosion rate in the steel bars. Chloride penetration depth was measured by spraying the surface of the sample after breaking it longitudinally. The chloride penetration depth in the cracked HPFRCC specimen was 25% lower than the reference mortar specimen. Similarly, the corrosion rate in HPFRCC was also reported to be 10% lower than the mortar specimen [45]. It was concluded that the durability in cracked FRC not only depends on the crack widths but also on the crack pattern which controls the macro- and micro-cell corrosion. Typically, a larger spacing between the cracks leads to the formation of a macro corrosion cell between the anode and cathode areas, and thus higher corrosion rates and mass loss of steel can occur [42]. On the other hand, with smaller spacing between the cracks, the movement of ions or charge is slow which forms microcell between the anode and cathode areas leading to less corrosion or mass loss of the steel bars. This hypothesis has been confirmed by Paul and van Zijl for SHCC specimens [62].

Corrosion resistance of HPFRCC as both patch repair and surface coating material was investigated by Kobayashi et al. [63]. Polyethylene (PE) fibers at dosages of 0.75%, 1%, and 1.5% by volume were used to prepare the HPFRCC. Three types of reinforced concrete (RC) beams: a monolithic beam made from normal concrete, beam with HPFRCC as a surface coating (in this case steel bars were inside a normal RC beam) and beam with HPFRCC as patch material (here steel bars were embedded into HPFRCC) were prepared and tested under chloride-induced corrosion. Cracks were generated in all specimens by pulling the steel bars using hydraulic jacks from both ends of the beams. In the RC beam, the maximum crack widths were between 0.36 mm to 0.65 mm. On the other hand, in the surface coated and patched HPFRCC specimens, multiple cracks were formed and the maximum widths were 0.01 mm to 0.12 mm. All specimens were then exposed to the 3% NaCl solution for 60 days by spraying the cracked face of the specimens for 5 min every 6 hrs. Chloride penetration and corrosion area of steel bars were then measured. No corrosion was observed in the beams with HPFRCC as a patch material even at a lower volume of fiber (0.75%). Also, chloride penetration was lower than in other cases. Beams with HPFRCC as coating material also showed an improvement compared to RC beams. Multiple fine cracks which formed in these HPFRCC specimens led to significant improvement in both corrosion resistance and chloride penetration resistance compared to RC specimens. Shaikh et al. [64] showed that the resistance of HPFRCC to chloride induced corrosion can be further improved when a hybrid mix of steel cord fibers and PE fibers is used. In this case, the corrosion mass loss and corrosion-induced longitudinal cracking were lower than in the HPFRCC specimens containing only PE fibers. It must also be noted that the accumulated chlorides in cracks due to wet-dry cycles, evaporation, and limited wash-out, increase the chloride concentration inside the crack. This may lead to similar chloride concentrations inside the crack faces compared to external exposed surfaces, i.e., the crack faces act as free surfaces [65]. 

In terms of practical applications, at present there is no agreement on the acceptable crack width threshold for chloride-induced corrosion limitation in FRC. Bernerd [66] concluded that crack widths greater than 0.10 mm lead to faster corrosion initiation than the uncracked state deterioration. A similar conclusion was also drawn in a study by Granju & Balouch [67], since they saw no signs of corrosion for specimens with crack widths lower than 0.1 mm even after one year of exposure to the marine-like environment. Self-healing of cracks can play a role, since it is known that small cracks have the ability to self-heal autonomously through so-called autogenous self-healing (i.e., without any external intervention or special additives) [68,69,70]. On the other hand, Mangat & Gurusamy [71] concluded that the permissible crack width in FRC is 0.20 mm. The tendency of higher corrosion activity was observed as the crack width in FRC was increased. However, Berrocal et al. [72] reported that the corrosion initiation is somewhat delayed for FRC made with both steel and PVA fibers, compared to reinforced concrete with the same surface crack width. Conversely, the electrical resistivity of concrete was reduced for both steel and PVA fiber FRC compared with RC. This is, of course, a major concern since lower electrical resistivity may ultimately result in higher corrosion rates [39]. Therefore, further investigations are required to address this issue.

It must be noted that the deterioration process (chloride ingress and corrosion) in FRC is significantly influenced by the fiber type. Some authors suggest that, while steel fibers themselves are certainly susceptible to corrosion, they have higher corrosion resistance due to the presence of millscale on the surface [73]. The durability of FRC with steel and macro-synthetic fibers was examined in pre-cracked specimens exposed to the coastal and inland environments for a duration ranges from 7 to 24 months [66]. It was found that the synthetic fibers themselves have excellent durability in both inland and coastal environment. In specimens with 0.20 mm crack width, steel fibers were significantly damaged by corrosion. The formation of pits at the crack bridging region of the steel fibers and the deformed areas leads to a significant reduction of the fiber cross-section and provokes notable reductions of the residual-tensile strength. Some research also suggested that the steel fibers with an inhibitor like Triethanolamine could be used to overcome the steel fiber corrosion problem of FRC [74]. Also, in a marine environment, FRC specimens made from steel fibers with crack widths below 0.1 mm were reported to be less vulnerable to corrosion [67]. In another study, the corrosion performance of FRC was investigated for three different types of fibers: melt extract (produced from stainless steel), corrosion-resistant, and low carbon steel [75]. After one year of marine exposure, FRC with melt extract and corrosion resistant fibers showed no sign of corrosion while significant corrosion was noticed in low carbon steel FRC. The reason for better corrosion resistance of melt extract and corrosion resistant fiber could also be their zinc-coated surface. Corrosion in steel fibers in FRC can also increase the roughness of the fibers, which may also increase the frictional bond in the fiber-matrix, thereby improving the residual tensile strength of FRC [67]. 

In general, the improved performance of FRC can be ascribed to the formation of more uniform chloride diffusion in multiple-cracked steel-reinforced FRC elements and structures, and a more uniform formation of smaller anode/cathode reaction compared to normal RC [76]. However, the large quantitative variations in the research results on FRC durability reported by different authors can be attributed to their different fiber contents, crack widths, exposure conditions and duration, fiber properties, concrete quality, etc. which might be reduced through systematic, comparative research efforts. The effect of fiber size on corrosion is not clear as it was suggested that the effect of wire lengths in the range of 0 to 160 mm can be negligible [77]. Moreover, controlling crack width in both NC and FRC is crucial in order to delay the corrosion initiation [78].

### 2.2. Fiber Reinforced Concrete Subjected to Carbonation

Carbonation causes chemo-mechanical changes in the concrete, in particularly changes in strength, permeability, pore size distribution, and chemistry. Furthermore, it is well-known that carbonation may cause shrinkage and thereby, potential cracking of the concrete [36]. It is also one of the prime deterioration factors that lead to corrosion of steel in RC structures [39]. This section reviews the available research data on the performance of FRC under carbonation attack.

From the literature, it is evident that the appropriate amount of fibers can delay the rate of carbonation in FRC compared to concrete without fibers. Since carbonation is related to the matrix porosity, pore structure and permeability, the optimum fiber content plays an important role in carbonation. The role of different percentages of steel fibers (0.0, 0.5, 1.0, 1.5 and 2%) on the carbonation depth of uncracked FRC was investigated by Wang et al. [79]. They reported that up to 1.5% fiber content can reduce the carbonation speed, while faster carbonation is reported at 2% fiber content. In another study, it was also found that 2% steel fiber content leads to higher porosity and permeability in FRC [80]. It must also be noted that higher porosity does not always result in higher permeability. The interconnectivity of the pores is more important [36,81]. However, regardless the fiber content, both the water permeability coefficient and gas permeability coefficient increase in FRC [80]. 

The performance of uncracked FRC specimens made with steel fibers subjected to carbonation was reported to be good, but some studies also reported severe corrosion damage in fiber at the bridging cracks which caused significant reduction in residual tensile strength of FRC [82,83]. In cracked FRC containing 0.5% PP fiber, self-healing of finer cracks during the test period also led to the reduction of CO_2_ penetration. Although no relation was found between the crack width and corrosion initiation time, a beneficial effect of fiber addition on carbonation induced corrosion rate was found [84]. Similarly, in another study of cracked FRC with low volume of steel fiber (0.6%) and polyester fibers (POL) (0.9%), carbonation depths were found to be 24% to 36% lower than control beams which were cracked at the same level of applied load [43]. Note that, as expected, the crack widths in control and FRC beams were different (0.24 mm for control and 0.10 mm for FRC), confirming the role of fibers in reducing the crack widths and thereby lowering the carbonation depth in FRC. Carbonation resistance of HPFRCC specimens made with 1.5% PE fiber and two different water-cement ratios (w/c) (0.30 and 0.60) was also investigated and compared with normal mortar specimens in cracked and uncracked states [45]. All specimens were exposed to 5% CO_2_ and the RH of 60% for 4 days and then a subsequent wet environment for 10 days with 90% RH to complete one cycle. In this way, a total of 6.5 cycles (about 91 days) were completed for specimens before measuring the carbonation depth. At lower w/c (0.30), carbonation depths in both uncracked HPFRCC and normal mortar specimens were almost the same (nearly zero). However, at higher w/c (0.6), the carbonation in HPFRCC specimens was more than double that of mortar specimens. An opposite trend was reported for cracked specimens. In this case, the penetration depth in mortar specimens was found to be three times higher than in the HPFRCC specimens. It must be noted that the residual crack width in mortar specimens was 0.3–0.4 mm, while in HPFRCC specimens it was 0.10 mm or less. Also, in mortar specimens, only a single crack was formed in contrast with the HPFRCC specimens where multiple cracks were formed. Therefore, the higher carbonation depth of mortar specimens can be attributed to the presence of deeper and wider cracks compared with HPFRCC specimens. Typically, carbonation develops faster at the cracked region and corrosion in the steel rebars at cracked regions is higher [26]. Also, in contrast with the microcell corrosion in chloride induced corrosion, macrocell prominent corrosion prevailed in carbonation induced corrosion [59]. The localized corrosion is generally fast for macrocell corrosion, while in microcell corrosion, this corrosion rate is slow [85]. Nevertheless, the reported data on the carbonation induced corrosion in FRC is limited.

The dense and uniform fiber-matrix interface of uncracked FRC creates a uniform coating surrounding the fibers, which may limit the access of oxygen and thus separate the steel rebars electrolyte (limiting the ion diffusion on rebar surface). This may ultimately delay the carbonation process at the steel and concrete interface [86]. In cracked FRC, the fiber–matrix interface can break, allowing oxygen and ions to be transported towards the steel surface. In this case, the steel fibers bridging the crack(s) act as anodes, and embedded steel rebars act as cathode due to the pH gradient at the crack region [87]. At the crack face, the pH is lower, since it allows access of water. If the crack widths are below a threshold level, self-healing may occur thus preventing the oxygen and ion transport. Cracks can heal due to the unhydrated cement particles, or be blocked by corrosion products, etc. in the crack region that would further limit the diffusion of oxygen and CO_2_. Also, if the cementitious matrix rich in Ca(OH)_2_, it may limit the decrease in pH and repassivate the anodic area [61].

### 2.3. Fiber Reinforced Concrete Subjected to Alkali-Silica Reaction (ASR)

Alkali-silica reaction (ASR) is a swelling reaction occurring between the highly alkaline cement paste and amorphous silica which may be present in the aggregates [88]. For ASR, a sufficient amount of moisture (above 80%) may also be present in certain aggregate types [89]. Generally, ASR produces a gel (also called ASR gel) which is soft, viscous and expansive in nature, produced from the sodium silicate [90]. ASR gel expands in the presence of water, causing pressure inside and around the siliceous aggregate, which may result in cracking and spalling, causing deterioration in the stiffness and strength of concrete [91,92]. Embedded fibers have the ability to bridge the cracks in concrete, motivating several researchers to investigate the ASR behavior in FRC. This section discusses the literature related to ASR in FRC.

**Figure 4:**
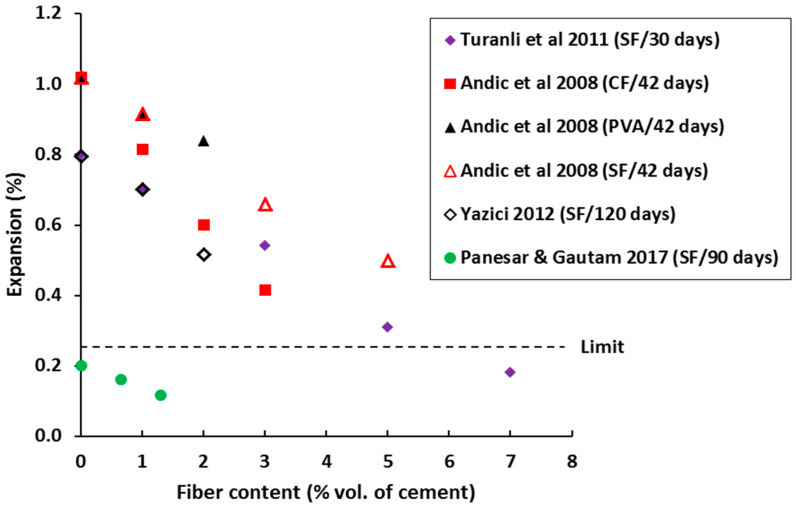
ASR expansion in FRC measured at different days for different fiber types and content (note: SF, CF and PVA means steel fiber, carbon fiber and PVA fibers) [88,93,95,96].

Several researchers investigated the effect of fiber inclusion in controlling the ASR induced cracking of concrete [93,94,95]. Most reports agree that the ASR expansion in FRC is lower than that of normal concrete without fibers (see Figure 4). Expansion was found to be reduced with the increased fiber content [93,96]. In FRC mortar mixes, micro-steel fibers were used at 0, 1, 3, 5 and 7% vol. of cement, respectively, and the expansion was measured every 24 h for 30 days as per ASTM C-1260 [96]. After 30 days of testing, expansion (%) was found to be 0.80 to 0.70, 0.55, 0.30, and 0.19 for fiber content of 0%, 1%, 3%, 5%, and 7%, respectively (see Figure 4). The reduction in expansion was even more pronounced for longer curing time, ascribed to the fact that the bond strength between the fibers and matrix increased. No influence of curing duration on the performance of the control specimen was observed. Higher tensile strength and small crack opening behavior of FRC containing micro-steel fibers not only limit the expansion of the ASR product, but also limits the migration of ASR gel away from the reaction site [97]. A similar conclusion of better expansion behavior of FRC with extended curing period and micro-fibers was also drawn in a study by Andic et al. [93]. Also, the extension of ASR cracks in FRC is dependent on the fiber type, content and age of the samples as shown in Figure 3. The dashed line in Figure 3 shows the maximum limit of aggregates expansion in a one-year concrete prism test as recommended by ASTM C 1293 [98]. Expansion above this considered to be the aggregates very highly reactive in nature.

**Figure 5:**
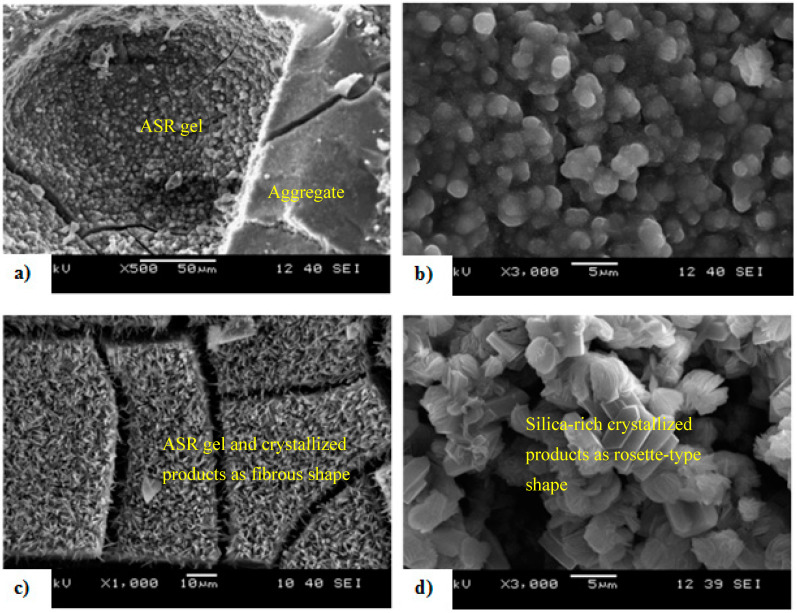
SEM image of (**a**) ASR product, (**b**) semi-organized products filling the pores in cement paste, (**c**) cracked products having a fibrous morphology and (**d**) a rosette-type morphology (adapted from [88]).

Effective ASR control in FRC was also reported at low volume content of steel fibers (1–2%) [88]. After submerging specimens for 120 days in NaOH solution at 80 °C, about 12% and 35% lower expansion was found for 1% and 2% micro steel fiber FRC, respectively, compared with reference concrete specimens. Scanning electron microscopy (SEM) was used to reveal the ASR products (silica, calcium and rich alkali) and their morphology (semi-organized, fibrous, rosette, etc.) in cracked FRC as shown in Figure 5 [88]. Energy dispersive X-ray analysis (EDX) showed that the calcium to silicate ratio (Ca/Si) and sodium to silicate ratio (Na/Si) in these products were higher, which determines the strength of cement paste. This reduced the inner deterioration of cracked steel fibers in ASR affected FRC. Furthermore, micro fibers are also effective in limitation of the deterioration in the mechanical properties caused by ASR. In general, the use of FRC is beneficial to extend the service life of structures due to its ability to preserve the residual mechanical properties and crack control capacity, even if significant damage processes take place [99,100,101].

### 2.4. Fiber Reinforced Concrete Exposed to High Temperature

In general, concrete reacts to high temperatures relatively well due to its incombustibility and low thermal diffusivity [102]. However, high temperatures in concrete do change its physical and chemical properties and affect the residual strength and fluid transport mechanism [103,104]. At high temperature (over 1000 °C), physiochemical changes of concrete may cause aggregate expansion, fractures on their crystalline microstructure, and even melting [102]. However, the deterioration of concrete properties is directly linked with the material and environment-related factors such as aggregate and cement types, w/c ratio, presence of fiber, exposure time, heating rate, etc. [102,105]. At high temperatures (above 400 °C), calcium hydroxide and calcium carbonate start to disintegrate. Similarly, calcium silicate hydrates also decompose after 500 °C and form pores in the microstructures [106,107]. 

The compressive strength of FRC with steel fiber gradually increases when the material is heated up to 200–300 °C, but starts to decrease as temperature further increases [108]. This is attributed to the improved hydration of unhydrated cement grains due to an internal autoclaving condition and the evaporation of water at high temperature [109]. FRC specimens with 2% PVA were exposed to different levels of temperature (20, 100, 200, 300, 400, and 600 °C) sustained for up to 2 and 6 h respectively before they were tested for tensile and compressive strength test after cooling down to room temperature [110]. It was revealed that no spalling occurred in FRC specimens even after 6 h of constant exposure to 600 °C (although their ductility was reduced significantly). In control specimens (mortar specimens without fibers), exposed to the same environment, severe spalling happened after 35–70 min. The better performance of FRC is attributed to its higher tensile capacity as well as increasing the porosity due to fibers melting. The formation of micro cracks was confirmed in the microstructure through SEM. The relatively low melting point of synthetic fibers (PP, PVA, PE) can play an important role in re-curing after exposed to high temperature. Fibers create micro channels in concrete which may accelerate water diffusion rate during water re-curing process [111]. However, steel fibers have a high melting point and can affect post heating mechanical behavior of FRC. The positive effect of steel fiber noticed just after cooling from high temperature, air re-curing of FRC can still restrict the formation of new cracks and Ca(OH)_2_ expansion [111]. Depending on the fiber type, the mechanical properties of FRC increased with temperature up to a certain limit (e.g., steel fiber 200–300 °C) due to fact that fibers inhibit the cracking growth that occurs in concrete at elevated temperature. And the optimum limit of temperature varies for fiber types and mix design. Combination of both steel and PP fiber (75% + 25%) also showed better performance than other combinations of (50% + 50%) and (25% + 75%) [112].

In another study, FRC was made with three different types of fiber (steel, PP, and PE) at 0.5 and 1.0 vol.% [113]. Prior to the flexural test, the specimens were exposed to high temperatures of 400, 600 and 800 °C in an oven according to ASTM E119-98. Up to 400 °C, all specimens showed increased flexural strength and toughness. For synthetic or plastic fibers (PP and PE) specimens, a significant difference was noticed at 800 °C compared with steel fiber specimens. The density of FRC measured by ultrasonic pulse velocity (UPV) also revealed that the mass loss in steel fiber specimens is lower than PP and PE fibers specimens [113]. Significant deterioration in FRC specimens made from steel fibers exposed to over 600 °C was also reported by Haddad et al. [114]. At high temperature, FRC with steel fibers may also change its behavior from brittle to pseudo-ductile and thus form multiple cracks in the structure before the failure occurs [115].

### 2.5. Fiber Reinforced Concrete Exposed to Freeze/Thaw Cycles

**Figure 6:**
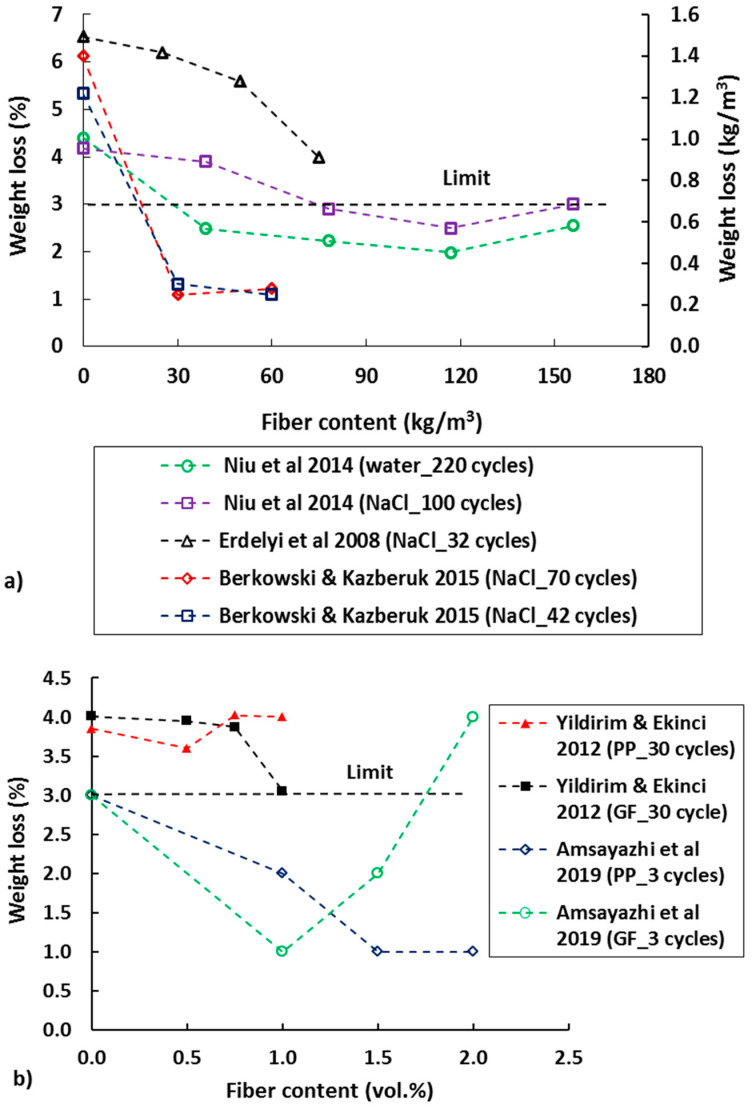
Weight loss of (**a**) steel-fiber FRC specimens and (**b**) polypropylene (PP) and glass fiber (GF) specimens’ solution under different freeze-thaw cycle (adapted from [120,121,122,123,124]).

The effect of fiber addition in concrete on reducing the freeze-thaw degradation in FRC is discussed in this section. The acceptable mass loss of concrete specimens (size 150 × 140 × 50 mm) in CDF (capillary suction of deicing chemicals and freeze-thaw test) test after 28 freeze-thaw cycles is <1500 g/m^2^ as recommended by the RILEM Committee TC 117-FDC45 [116]. Similarly, for a test involving 56 freeze-thaw cycles in 100 mm cubes, the acceptance criterion is <3% by weight of scaled material as recommended in [117]. As indication of deterioration, the percentages of weight loss of FRC specimens with different steel fiber contents and solutions (water and NaCl) under freeze-thaw exposures have been studied by several researchers (see Figure 6). The weight loss of the FRC specimens was reduced as the steel fiber content increased (see Figure 6a). The w/c was also found to influence the weight loss of the specimens [118,119]. It was found that the ultimate freeze-thaw cycles (i.e., specimens cracked and removed from the freeze-thaw chamber) in FRC specimens with lower w/c (0.26) was 1900, while the specimens with w/c of 0.32 and 0.44 sustained (before they were removed from the testing chamber) only 780 and 260 cycles, respectively. Even the mass loss was significantly lower at low w/c compared to higher w/c. The decline in dynamic modulus of elasticity in FRC specimens was slower than the control specimen. This is attributed to the crack arresting behavior of FRC. The damage in the concrete specimens depends on their ability to accommodate the increased volume of frozen solution in the pores. Fiber types also play an important role, as shown in Figure 1b. For PP and glass fiber (GF) the optimum limit of fiber content seems to be below 2%.

**Figure 7:**
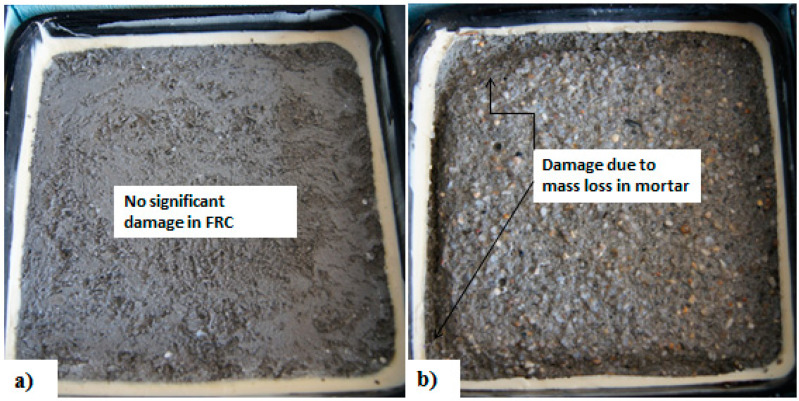
Damage on the surface of (**a**) FRC with PVA fiber and (**b**) high strength mortar specimen after 28 freeze-thaw cycles.

Excellent resistance of FRC with synthetic fibers to freezing and thawing has also been reported in many studies [3]. Yin et al. [125] reported that the FRC with PVA fibers had no noticeable mass loss even after 500 freeze-thaw cycles. However, the flexural strength of FRC did decrease with the number of cycles. Analysis of the microstructure using SEM revealed that, while needle-like ettringite crystals can be found in FRC samples before freeze-thaw cycles, they gradually disappeared and the internal structure of FRC became more compact with freeze-thaw exposure. However, the internal structure was damaged due to freeze-thaw attack and micro cracks gradually formed, leading to reduced flexural strength as the number of freeze-thaw cycles increased [93]. Crack bridging ability of micro fibers FRC specimens also can minimize the interconnecting pores in the matrix and thus stop the ice formation in the pores, leading to less damage in the specimens. In general, freeze-thaw cycles deteriorate and weaken the fiber-matrix interface in FRC [126]. Better resistance of FRC with low volume (0.1%) of PP compared to a control mix was also found by Wang & Chen [127]. As for steel fibers, mass loss in synthetic FRC specimens due to freeze/thaw cycles was also reduced as the fiber content increased [122]. Figure 7 shows a comparison of SHCC with 2% PVA and mortar specimens’ performance after 28 freeze-thaw cycles. Significantly higher mass loss was measured in the mortar (28 days compressive strength 62 MPa) compared to SHCC with significantly lower compressive strength (compressive strength 30 MPa).

Compared to synthetic, steel fibers appeared to be more effective for freeze-thaw resistance. Also, straight short fibers have shown better efficiency than longer hooked ends fibers [120]. This can be explained by the number of fibers per unit volume of materials and in the surface layer. In general, the number of longer, hooked end fibers per volume is lower compared to straight fibers. Therefore, a proper selection of fiber type, size, and content can play an important role of the concrete damage due to frost action.

## 3. Remaining Service Life of FRC Infrastructures

**Figure 8:**
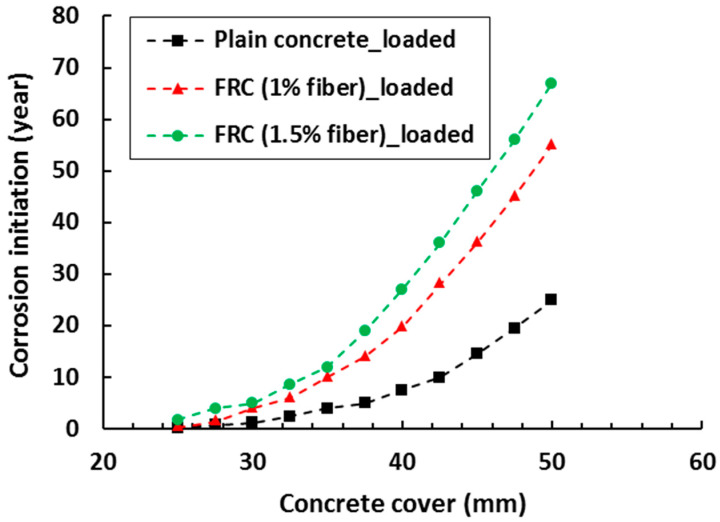
Predicted corrosion initiation of FRC and plain concrete at varying cover depth (adapted from [128]).

This section discusses the existing models that deal with FRC especially in the area of corrosion. In a study by Wang et al. [128], time to corrosion initiation of FRC was evaluated using Fick’s second law as shown in Figure 8. The authors note that, although Fick’s second law is not able to physically represent the variety of mechanisms that lead to chloride ingress in concrete (e.g., capillary suction, wick action, wetting and drying cycles, etc.), it is the most widely used approach in the scientific literature and engineering practice due to its simplicity [41]. It was found that the benefit of FRC over RC under unloading condition is not obvious as the difference in results was insignificant. However, under the same loading condition (bending load), time to corrosion initiation in FRC was prolonged about 2.2 to 3.6 times; varying with the fiber content and cover thickness (see Figure 8). In a study of Dhinakaran et al. [129], a comparison of life cycle cost between glass fiber reinforced polymer (GFRP) and normal RC beams was performed. It is concluded that the beam made with GFRP is about 40% more economical and also has two times longer service life compared to the RC beam. The improved performance of FRC can be attributed to its crack bridging and multiple cracking behaviors which control the macro and micro-corrosion.

**Figure 9:**
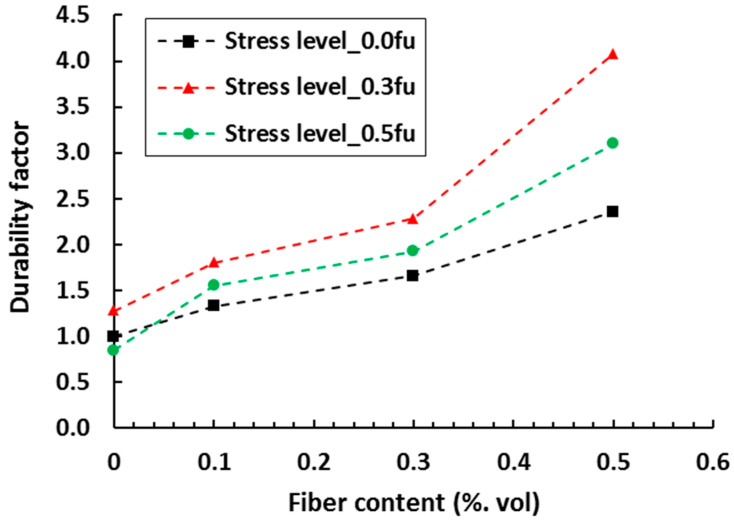
Influence of fiber content and stress level on the durability factor of plain and FRC specimens. Here f_u_ represent the ultimate compressive strength of concrete (adapted from [130]).

The expected service life of FRC specimens made from different percentages of softwood fiber (0.1, 0.3 and 0.5%) with and without applied compressive stress was predicted for the chloride diffusion coefficients as shown in Figure 9 [130]. The diffusion coefficient was estimated using an empirical relation [131] and then used to estimate the service life. Finally, the durability factor, D for a given concrete was defined as the ratio of its expected service life to that of companion plain concrete under zero stress. The durability factor found to be highest at the compressive stress level of 0.3 times of ultimate compressive strength (0.3 f_u_). In another study [132], the service life of SHCC was predicted from an accelerated corrosion mass loss testing for different cover depths (15, 25, and 35 mm). Here the corrosion mass loss of steel rebars was determined by applying different voltages (10, 20 and 30 V) up to a certain time period while specimens were exposed to the chloride solution. Corrosion mass loss was translated to the actual loss in resistance for service life estimation by a determination of the mass loss distribution. Acceleration factors which define the deterioration time of the SHCC specimens for different cover depths were then determined from an Arrhenius-type relation.

Studies were also devoted to develop service life models of FRC for temperature and freeze-thaw actions and, in both cases, increased life for FRC compared to plain concrete was found [133,134]. Under the same freeze-thaw testing condition, the service of FRC specimens (with 1% fiber) was 11 years longer than conventional concrete [133]. The proposed models may be useful when designing structures such as water tanks, bridge decks, and marine structures [130]. Nonetheless, the drawback of all service life predictions is that they are based on laboratory tests, and real deterioration conditions may be very different. Monitoring of long-term deterioration under natural conditions is therefore required to calibrate the acceleration factors determined at an earlier stage [135]. Also, service life prediction models could be updated by obtaining more field data using sensors or on-site corrosion surveys which are fed to the probabilistic prediction models rather than relying on laboratory experiments [136].

## 4. Durability of Existing FRC Infrastructures

Traditional FRC applications have seen a distinction between use of micro-fiber and macro-fiber FRCs. Micro-fiber FRCs have been widely used for plastic shrinkage control [137,138], impact resistance [139,140,141,142], and fire resistance [143,144]. Macro fiber FRCs have been applied in structural applications such as slabs on grade as industrial floors and road pavements [145], to a lesser degree in suspended slabs [146,147] and tunnel linings [148,149]. Recent development of advanced classes of FRC has also resulted in the use of short, fine micro-fibers in structural applications. In this section, the durability performance of various FRC infrastructures is reported, first in conventional applications, and subsequently in retrofitting/rehabilitation of infrastructures with advanced classes of FRC.

### 4.1. Performance of Traditional FRC Infrastructures—Ground and Suspended Slabs

The dominant conventional FRC application was reported to be slabs on grade, or so-called ground slabs in the form of industrial floors and road pavements [150], which comprised about 60% of FRC applications. Fibers are dominantly used for crack control as secondary reinforcement [145], which may lead to significant life cycle cost saving by reduced cost for maintenance and repair due to wheel impacts and spalling at floor and pavement joints or shrinkage cracks in jointless slabs. These types of applications have also progressed to design guidelines, e.g., Technical Report 34 of the Concrete Society [151], fib Model Code [152], ACI 360 [153], although no standardized design is yet included in Eurocode or ACI Concrete codes.

In suspended slabs, the fibers contribute to crack width reduction and crack spacing control, which improves serviceability and durability [152]. However, the use of fibers as the only reinforcement (i.e., without conventional steel rebars) in suspended slabs has been limited. A general belief is that a combination of traditional steel bar reinforcement and fibers is required for reliable structural behavior, and it has recently been suggested that an appropriate combination of reinforcement bars and fibers may yield the lowest total reinforcement ratio [154]. Yet, suspended slabs reinforced only with fibers have been constructed in a number of commercial and residential buildings in Baltic countries, continental Europe and the UK, including a five-story office building and a sixteen-story office tower [146,147]. The thin slabs span 5–8 m and contain steel fibers at volume percentages in the range of 0.6–1.2% (45–100 kg/m^3^). However, steel bar reinforcement was incorporated in the strips spanning between columns to address the risk of progressive collapse. At the time of publication, no reports were received on the performance of these slabs from the authors. However, successful and routine use of shorter span (3–5 m) suspended FRC (only) slabs for a period of 15 years has been reported [146]. In the indoor environment, the main deterioration mechanism of FRC slabs is believed to be carbonation and carbonation-induced corrosion due to the exposure to occupant or industry-related sources of CO_2_. As reported in Section 2.2, an optimal FRC mix design can reduce the rate at which the carbonation front progresses deeper into the concrete. However, no reports on such deterioration related to practical applications of FRC have been reported.

### 4.2. Performance of Traditional FRC Infrastructures—Tunnel Linings

While FRC tunnel linings were introduced in the 1980s [155], the first FRC tunnel lining design guidelines are the ACI 544.7R [153] from 2016. Shotcrete, precast or cast in-situ FRC tunnel linings have all been used. Deterioration processes in tunnel linings are reported to include chloride and carbonation-induced corrosion, sulfate and acid attack, freeze-thaw, ASR, and in railway tunnels stray current corrosion [156,157]. Discrete steel fibers in FRC have been found to be less susceptible to stray-current corrosion than RC, mainly by the discontinuous nature of the dispersed fibers [142]. The performance of FRC linings subjected to fatigue by repetitive loading through air pressure and suction from passing trains depends on prevention of cracks. In absence of cracks, fatigue life of FRC linings are superior to that of plain concrete, but in cases where restrained shrinkage does lead to crack formation, cyclic loading may cause wide cracks with high risk of reduced fatigue life [158]. Chloride-induced corrosion of steel fibers themselves has been reported in Section 2.1 to be less significant in cracked steel FRC than for steel bars, with no or insignificant corrosion on fibers bridging cracks less than 0.1 mm wide, and light corrosion with no loss in cross section of fibers in wider (0.5 mm) cracks [67].

### 4.3. Infrastructure Retrofitted with FRC

Aging of infrastructure, damage by earthquakes and premature corrosion of reinforcement in coastal and regions where de-icing salts are used have led to use of FRC strengthening and retrofitting interventions on highway infrastructure. The performance of retrofitted infrastructure for improved resistance to ingress of water and deleterious substances by the use of high density FRC, as well as where greater deformability and ductility have been provided, is reported here.

Ultra-high strength (180–200 MPa compressive and 7–15 MPa tensile strength) UHPFRC with low water permeability has been applied in partial replacement of deteriorated decks and slabs of several road and railway infrastructures and buildings since 2004 [159]. The high strength and low permeability of these materials combine functions of strengthening and resistance to ingress of deleterious substances, thereby impairing deterioration processes [5]. Typically, 25–40 mm thick UHPFRC bonded overlays containing high strength steel fibers or HMPE fibers have been applied after removal of deteriorated concrete. The thin overlays restored or increased resistance of the superstructures without adding significant weight and the need to strengthen substructures. The interventions were to deteriorated infrastructures aged 50–70 years, restoring service life expectancy to the typical 100 years for such infrastructure.

**Figure 10:**
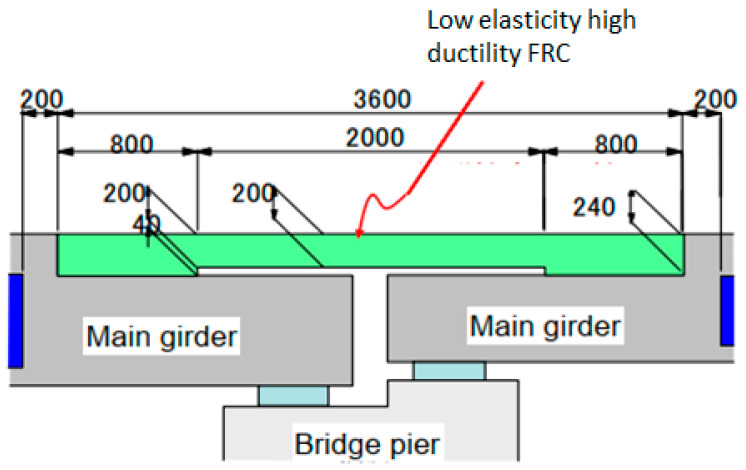
Schematic of a bridge deck connecting slab (adapted from [160]).

To improve earthquake resistance and reduce maintenance requirements of movement joints in highway bridges, a slab connecting system has been developed [160,161]. The concept is illustrated in Figure 10. Highly ductile SHCC was used for the connecting slab, with a nominal compressive strength of 30 MPa and an elastic modulus of 15–20 GPa. The ductility was designed for multiple crack formation in the connecting slab which may occur due to shrinkage or an earthquake, whereby relative movement between the two girders is accommodated. Waterproofing and asphalt are placed over the connecting slab to ensure the geometrical continuity. This means that the condition of the R/SHCC connecting slab cannot be inspected visually. However, it appears to be in good condition due to a lack of visible cracks or potholes on the asphalt since its installation in 2011.

**Figure 11:**
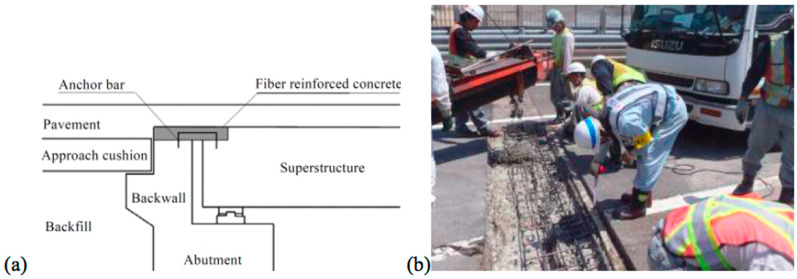
Schematic of an (**a**) FRC abutment-slab connection to prevent leakage and corrosion in highway infrastructure, and (**b**) casting of the FRC [162].

In another retrofit to improve infrastructure durability with FRC, Ishikawa et al. [162] developed an abutment-slab connection illustrated in Figure 11. Significant leakage and associated corrosion of reinforced concrete in the abutment and slabs under traditional joints in highway bridges motivated this intervention. Ishikawa et al. [162] reported to have performed 85 joint replacements using this approach. In addition, the approach allows that waterproofing and asphalt can be placed continuously over the connecting FRC joint. Upon inspection, delamination was found in three cases, which led to leakage, but in addition to significantly reduced leakage, vehicle wheel impact is also reduced by this jointless link compared with traditional joints. Hence, in addition to improved durability, the intervention resulted in improved driving comfort and fatigue life of these bridges.

**Figure 12:**
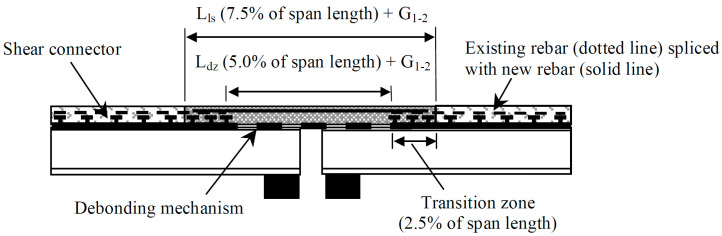
Schematic of a link slab replacing a problematic movement joint in multi-span highway bridges subject to de-icing salt deterioration (adapted from [163]).

Figure 12 illustrates a third intervention to improve durability of highway bridges by FRC retrofitting, the so-called link slab concept [163,164]. Traditional movement joints that allow leakage of water and de-icing salts, and associated corrosion damage in the substructure or deck, are replaced with highly ductile, steel reinforced SHCC. The replacement of traditional movement joints was recorded to be as frequent as every three years. However, a link slab constructed in 2005 was reported to still be operational and functioning well. Multiple cracks are visible on the link slab surface at regular spacing, but controlled by fiber reinforcement to prevent localization in wide cracks and associated accelerated deterioration.

## 5. Concluding Remarks

In this paper, the properties of different types of FRC exposed to various deterioration processes are reviewed. The mechanisms of improved durability brought by the addition of fibers are elaborated, as are pitfalls causing negative impacts on concrete durability. The authors believe that this review may bring insight and perspective to researchers and designers on the durability potential and performance of FRC, and of FRC used in combination with reinforcing steel bars, enabling them to select these materials for appropriate applications confidently. The following conclusions are drawn from the review:

- A main durability enhancing feature of FRC is crack control which limits the rate at which deleterious substances such water, chlorides, and carbon dioxide ingress into structural elements, thereby prolonging the service life of the structure.
- There is evidence that also the propagation period is prolonged in R/FRC compared with RC. This applies to most dominant deterioration processes in infrastructures, which are carbonation and chloride-induced corrosion, and ASR-deterioration.
- Fibers in concrete may alter and improve resistance to other deterioration mechanisms. Corrosion induced by stray current in rail-infrastructure is reduced or prevented by the dispersed, non-interconnected fiber, compared with continuous steel bar reinforced structures. Polymeric fiber in FRC reduces of prevents explosive spalling in situations of fire, by melting and freeing up void space for expansion.
- As in RC, wide cracks in FRC or R/FRC lead to high deterioration rates. A typical threshold crack width is 0.1 mm. Crack widths in excess expose crack surfaces to ingress and subsequent high deterioration rates. Apart from low ingress and deterioration rates for fine cracks, self-healing of cracks up to this threshold may occur, further reducing deterioration processes.
- It is possible and essential to combine improved durability of FRC with enhanced mechanical integrity. Structural systems like link/connecting slabs on bridge infrastructure are excellent examples of efficient use of customized FRC design, combining sufficient deformability and crack control by fiber bridging toward medium- and long-term cost-efficiency by avoiding regular maintenance and repair interventions and safety in seismic events.

Deterioration mechanisms in FRC are not yet fully understood or characterized. The quantification of deterioration rates in actual structures in the field and appropriate limit states of durability behavior are of crucial importance. Even for RC, durability design that includes both initiation and propagation phases of reinforcement corrosion is not yet established, although more information is available. An added complexity is significantly reduced crack spacing potential with particular FRC classes, for which there is evidence that deterioration processes are altered and their rates reduced, for instance by predominant micro-cell corrosion in multiply and finely cracked FRC, as opposed to high-rate macro-cell corrosion in wider, localized cracks in RC. To fully exploit the durability and service life preservation potential by appropriate FRC material and structural design, research effort towards fully understanding these mechanisms, increased data pools, and standardization are required. Finely, standardized durability tests are imperative, that enable characterization of the durability performance of structures in the field. Long duration durability tests in the field are vital, to enable not only improved durability of infrastructures, but improved predictability toward reliable service life design.

## Figures and Tables

**Table 1:** Physical properties of commonly used fiber types in FRC.

Types of Fibers	Diameter (µm)	Length (mm)	Specific Gravity (g/cm^3^)	Tensile Strength (MPa)	Elastic Modulus (GPa)	Ultimate Elongation (%)
Steel	5–1000	10–60	7.85	200–2600	195–210	0.5–5.0
Polyethylene (PE)	25–1000		0.96	80–600	1.4–5	12–100
High modulus PE (HMPE)	20–24	6–12	0.97	2500–3000	80–120	2.5–5
As-spun phenylene-bensobisoxazole (PBO-AS)	13	6	1.54	5800	180	3.5
Polypropylene (PP)	10–200	5–50	0.90–0.91	310–760	3.5–14.7	6–15
Polyvinyl alcohol (PVA)	9–760	6–12	1.2–2.5	800–3600	20–80	4–12
Glass	6–35	10–50	2.54–2.70	1500–4000	72–80	2.5–4.8
Coconut	100–400	–	1.12–1.15	120–200	19–25	10–25
Jute	100–200	–	1.02–1.04	250–350	25–32	1.5–1.9
Asbestos	0.02–25	5–40	2.55–3.2	200–1800	164	2–3
Carbon	7–20	3–12	1.2–2	600–4000	200–390	0.4–11

**Table 2:** A general overview of the influence of cracks in FRC on the corrosion of reinforcing steel. Note that *W_cr,ave_* is the average crack width, while *W_cr,total_* is the total crack width.

Reference	Fibers Type/Content	Exposure Time (month)	Crack Width (mm)	Carbonation Depth (mm)	Total Chloride Content (%)	Corrosion Damage
Vasanelli et al. [43]	Steel, 0.6%	17	*W_cr,ave_* 0.13	15.11–16	–	–
	Polyester fibers, 0.9%	17	*W_cr,ave_* 0.13–0.16	14.87–17.47	–	–
Kobayashi & Kojima [44]	Polypropylene, 1.5%	11	*W_cr,tot_* 0.04–0.26	–	0.38–0.63	Mass loss, 5%
	Polypropylene, 1.0%	11	*W_cr,tot_* 0.02–0.12	–	0.55–0.62	Mass loss, 8%
	Polypropylene, 0.75%	11	*W_cr,tot_* 0.04–0.08	–	0.55–0.60	Mass loss, 10%
Miyazato & Hiraishi [45]	Polyethylene, 1.5%	3	*W_cr,ave_* < 0.1	21.6 (w/c = 0.3) 28.8 (w/c = 0.6)	–	–
Micelli et al. [46]	Steel, 0.6%	72	*W_cr,ave_* 0.19	39	–	–
	Polyester fibers, 0.9%	72	*W_cr,ave_* 0.16	37	–	–
Chen et al. [47]	Steel, 0.5%	36	*W_cr,ave_* 0.10–0.40	–	–	Pitting area, ~17%
	Polyvinyl alcohol, 0.75%	36	*W_cr,ave_* 0.10–0.40	–	–	Pitting area, ~20%

## Nomenclature

ASR	Alkali-silica reaction
CDF	Capillary suction, Internal damage and Freeze-thaw test
ECC/SHCC	Engineered, strain hardening cementitious materials
EDX	Energy dispersive x-ray spectroscopy
FRC	Fiber reinforced concrete
GF	Glass fiber
HMPE	High modulus polyethylene
HPFRC	High performance fiber-reinforced concrete
HPFRCC	High-performance fiber-reinforced cementitious composite
PE	Polyethylene
PP	Polypropylene
PVA	Polyvinyl alcohol
RCPT	Rapid chloride migration test
SEM	Scanning electron microscopy
UHPFRC	Ultra-high performance fiber-reinforced concrete
